# Stealing PINs via mobile sensors: actual risk versus user perception

**DOI:** 10.1007/s10207-017-0369-x

**Published:** 2017-04-07

**Authors:** Maryam Mehrnezhad, Ehsan Toreini, Siamak F. Shahandashti, Feng Hao

**Affiliations:** School of Computing Science, Newcastle University, Newcastle upon Tyne, UK

**Keywords:** Mobile sensors, JavaScript attack, Mobile browsers, User security, User privacy, Machine learning, PINs, Risk perception, User study

## Abstract

In this paper, we present the actual risks of stealing user PINs by using mobile sensors versus the perceived risks by users. First, we propose PINlogger.js which is a JavaScript-based side channel attack revealing user PINs on an Android mobile phone. In this attack, once the user visits a website controlled by an attacker, the JavaScript code embedded in the web page starts listening to the motion and orientation sensor streams without needing any permission from the user. By analysing these streams, it infers the user’s PIN using an artificial neural network. Based on a test set of fifty 4-digit PINs, PINlogger.js is able to correctly identify PINs in the first attempt with a success rate of 74% which increases to 86 and 94% in the second and third attempts, respectively. The high success rates of stealing user PINs on mobile devices via JavaScript indicate a serious threat to user security. With the technical understanding of the information leakage caused by mobile phone sensors, we then study users’ perception of the risks associated with these sensors. We design user studies to measure the general familiarity with different sensors and their functionality, and to investigate how concerned users are about their PIN being discovered by an app that has access to all these sensors. Our studies show that there is significant disparity between the actual and perceived levels of threat with regard to the compromise of the user PIN. We confirm our results by interviewing our participants using two different approaches, within-subject and between-subject, and compare the results. We discuss how this observation, along with other factors, renders many academic and industry solutions ineffective in preventing such side channel attacks.

## Introduction

Smartphones equipped with many different sensors such as *GPS, light, orientation, and motion* are continuously providing more features to end users in order to interact with their real-world surroundings. Developers can have access to the mobile sensors either by (1) writing native code using mobile OS APIs [1], (2) recompiling HTML5 code into a native app [2], or (3) using standard APIs provided by the W3C which are accessible through JavaScript code within a mobile browser.^1^ The last method has the advantage of not needing any app-store approval for releasing the app or doing future updates. More importantly, the JavaScript code is platform independent, i.e., once the code is developed it can be executed within any modern browser on any mobile OS.

**Fig. 1:**
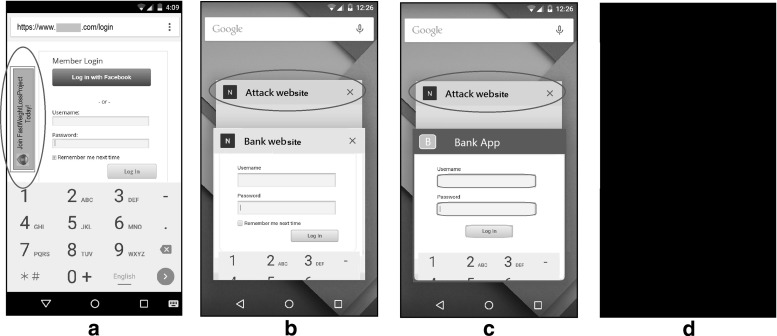
PINlogger.js potential attack scenarios; **a** the malicious code is loaded in an iframe and the user is on the same tab, **b** the attack tab is already open and the user is on a different tab, **c** the attack content is already open in a minimized browser, and the user is on an installed app, **d** the attack content is already open in a (minimized) browser, and the screen is locked. The attacker listens to the side channel motion and orientation measurements of the victim’s mobile device through JavaScript code, and uses machine learning methods to discover the user’s sensitive information such as activity types and PINs

*In-browser access risks* While sensor-enabled mobile web applications provide users more functionalities, they raise new privacy and security concerns. Both the academic community and the industry have recognized such issues regarding certain sensors such as geolocation [3]. For a website to access the geolocation data, it must ask for explicit user permission. However, to the best of our knowledge, there is little work evaluating the risks of in-browser access to other sensors. Unlike in-app attacks, an in-browser attack, i.e., via JavaScript code embedded in a web page, does not require any app installation. In addition, JavaScript code does not require any user permission to access sensor data such as device motion and orientation. Furthermore, there is no notification while JavaScript is reading the sensor data stream. Hence, such in-browser attacks can be carried out far more covertly than the in-app counterparts.

However, an effective in-browser attack still has to overcome the technical challenge that the sampling rates available in browser are much lower than those in app. For example, as we observed in [4], frequency rates of motion and orientation sensor data available in-browser are 3 to 5 times lower than those of accelerometer and gyroscope available in-app.

*In-browser attacks* Many popular browsers such as Safari, Chrome, Firefox, Opera, and Dolphin have already implemented access to the above sensor data. As we demonstrated in [5] and [4], all of these mobile browsers allow such access when the code is placed in any part of the active tab including *iframes* (Fig. 1a). In some cases such as Chrome and Dolphin on iOS, an inactive tab can have access to the sensor measurements as well (Fig. 1b). Even worse, some browsers such as Safari allow the inactive tabs to access the sensor data, when the browser is minimized (Fig. 1c), or even when the screen is locked (Fig. 1d).

Through experiments, we find that mobile operating systems and browsers do not implement consistent access control policies in regard to mobile orientation and motion sensor data. Partly, this is because W3C specifications [6] do not specify any policy and do not discuss any risks associated with this potential vulnerability. Also, because of the low sampling rates available in browser, the community have been neglecting the security risks associated with in-browser access to such sensor data. However, in TouchSignatures [4], we showed that despite the low sampling rates, it is possible to identify user touch actions such as click, scroll, and zoom and even the numpad’s digits. In this paper, we introduce PINLogger.js, an attack on full 4-digit PINs as opposed to only single digits in [4].

*Mobile sensors* Today, sensors are everywhere: from your personalized devices such as mobiles, tablets, watches, fitness trackers, and other wearables, to your TV, car, kitchen, home, and to the roads, parking lots, and smart cities. These new technologies are equipped with many different sensors such as NFC, accelerometer, orientation, and motion and are connected to each other. These sensors are continuously providing more features to end users in order to interact with their real-world surroundings. While the users are benefiting from richer and more personalized apps which are using these sensors for different applications such as fitness, gaming, and even security application such as authentication, the growing number of sensors introduces new security and privacy risks to end users, and makes the task of sensor management more complex.

*Research questions* While sensors on mobile platforms are getting more powerful and starting to collect more information about the users and their environment, we want to evaluate the general knowledge about these sensors among the mobile users. We are particularly interested to know the level of concern people may have about these sensors being able to threaten their privacy and security.

*Contributions* In this work, we contribute to the study of sensors and their actual risks and their perceived risks by users as follows:

- We introduce PINLogger.js, an attack on full 4-digit PINs as opposed to only single digits in [4]. We show that unregulated access to these sensors imposes more serious security risks to the users in comparison with more well-known sensors such as camera, light, and microphone.
- We conduct user studies to investigate users’ understanding about these sensors and also their perception of the security risks associated with them. We show that users in fact have fewer security concerns about these sensors comparing to more well-known ones.
- We study and challenge current suggested solutions, and discuss why our studies show they cannot be effective. We argue that a usable and secure solution is not straightforward and requires further research.

**Fig. 2:**
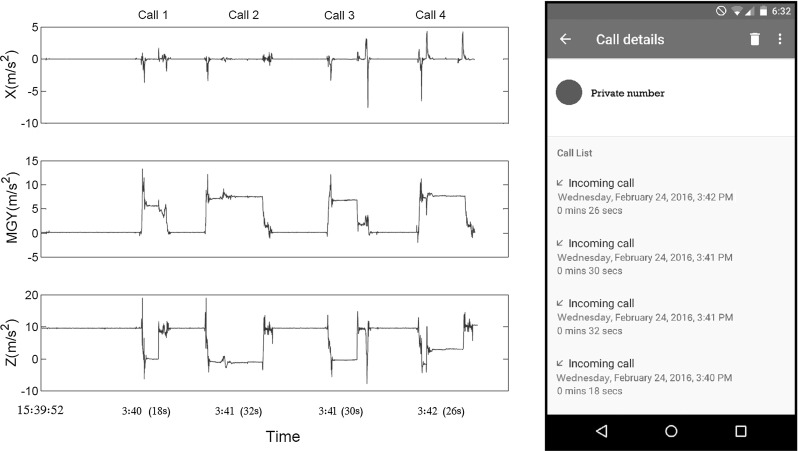
*Left* Three dimensions (*x*, *y*, and *z*) of acceleration data including gravity (from the motion sensor). The start time, duration, and end time of four phone calls are easily recognizable from these measurements. *Right* The screenshot of the call history of the phone during the experiment

## User activities

The potential threats to the user security posed by an unauthorized access to the motion and orientation sensor data are not immediately clear. Here we demonstrate two simple scenarios which show that sensitive user information such as phone calls timing and physical activities can be deduced from device orientation and motion sensor data obtained from JavaScript.

Users tend to move their mobile devices in distinctive manners while performing certain tasks on the devices, or by simply carrying them. Examples of the former include answering a call or taking a photograph, while the latter covers their transport mode. In both cases, an identifiable succession of movements is exhibited by the device. As a result, a web-based program which has access to the device orientation and motion data may reveal sensitive facts about users such as the exact timing information of the start and end of phone calls and that of taking photographs. On the other hand, while the user is simply carrying her device, the device movement pattern may reveal information about the user’s transport mode, e.g. if the user is stationary at one place, walking, running, on the bus, in a car or on the train. We present the results of two initial experiments that we have performed on a Nexus 5 using *Maxthon Browser* (as an example of a browser that allows JavaScript to access sensor data even when the screen is locked).

*Motion and orientation sensors detail* Before, presenting the results, we first explain the motion and orientation sensors in detail. According to W3C specifications [6], motion and orientation sensor data are a series of different measurements as follows:

- device *orientation* which provides the physical orientation of the device, expressed as three rotation angles ($\alpha$, $\beta$, $\gamma$) in the device’s local coordinate frame,
- device *acceleration* which provides the physical acceleration of the device, expressed in Cartesian coordinates (*x*, *y*, *z*) in the device’s local coordinate frame,
- device *acceleration including gravity* which is similar to acceleration except that it includes gravity as well,
- device *rotation rate* which provides the rotation rate of the device about the local coordinate frame, expressed as three rotation angles ($\alpha$, $\beta$, $\gamma$), and
- *interval* which provides the constant sampling rate and is expressed in milliseconds (ms).

The device coordinate frame is defined with respect to the standard position of the mobile screen. When it is in the portrait mode, *x* and *y* axes are in the plane of the screen and are positive towards the screen’s right and up, and *z* is perpendicular to the plane of the screen and is positive outwards from the screen. Moreover, the sensor data discussed above are processed sensor data obtained from multiple physical sensors such as gyroscope and accelerometer. In the rest of this paper, unless specified otherwise, by sensor data we mean the sensor data accessible through mobile browsers which include acceleration, acceleration including gravity, rotation rate, and orientation.

*Phone call timing* In the first experiment, we opened the website carrying our JavaScript code and then locked the screen. The JavaScript code continued to log orientation and motion data while the Android phone was left on a desk. For this experiment, we used another phone to call the Android phone four times with a few seconds apart between the calls. As demonstrated in Fig. 2 (left), the 4 distinct phone calls along with their timing are recognizable from the three dimensions of acceleration (including gravity) which come from the device motion sensor. For a better comparison, Fig. 2 (right) shows the received call history of the phone during the experiment with their start times and durations. As shown in this figure, the captured sensor data match the call history.

**Fig. 3:**
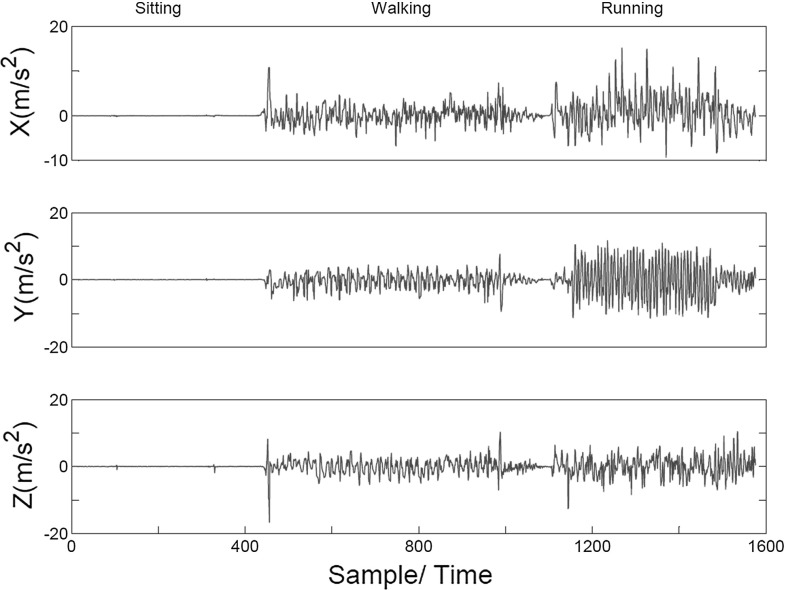
Three dimensions (*x*, *y*, and *z*) of acceleration data (from the motion sensor) during 22 s of sitting, 34 s of walking and 25 s of running

*User physical activities* In the second experiment, we again locked the phone and recorded the sensor data during 22 s of sitting, 34 s of walking and 25 s of slow running. We observed that the mentioned activities have visibly distinctive sensor streams. As an example, Fig. 3 shows the acceleration data from motion sensor. As it can be seen, the mentioned activities are recognizable from each other since they are visibly different in the sensor measurements.

Our initial evaluations suggest that discovering device movement related information such as call times and user’s mode of transport can be easily implemented. However, as we will explain, distinguishing user PINs is a lot harder as the induced sensor measurements are only subtly different. In the following sections, we will demonstrate that, with advanced machine learning techniques, we are able to remotely infer the entered PINs on a mobile phone with high accuracy.

## PINlogger.js

In this section, we describe an advanced attack on user’s PINs by introducing PINlogger.js. In the following subsections, we describe the attack approach, our program implementation, data collection, feature extraction, and neural network.

### Attack approach

We consider an attacker who wants to learn the user’s PIN tapped on a soft keyboard of a smartphone via side channel information. We consider (digit-only) PINs since they are popular credentials used by users for many purposes such as unlocking phone, SIM PIN, NFC payments, bank cards, other banking services, gaming, and other personalized applications such as health care and insurance. Unlike similar works which have to gain the access through an installed app [7–16], our attack does not require any user permission. Instead, we assume that the user has loaded the malicious web content in the form of an iframe, or another tab while working with the mobile browser as shown in Fig. 1. At this point, the attack code has already started listening to the sensor sequences from the user’s interaction with the phone.

In order to uncover when the user enters his PIN, we need to classify his touch actions such as click, scroll, and zoom. We have already shown in TouchSignatures [4] that with the same sensor data and by applying classification algorithms, it is possible to effectively identify user’s touch actions. Here, we consider a scenario after the touch action classification. In other words, our attacker already knows that the user is entering his PIN. Moreover, unless explicitly noted, we consider a generic attack scenario which is not user dependant. This means that we do not need to train our machine learning algorithm with the same user as the subject of the attack. Instead, we have a one-round training phase with data from multiple voluntary users and use the obtained trained algorithm to output other users’ PINs later. This approach has the benefit of not needing to trick individual users to collect their data for training.

### Web program implementation

**Fig. 4:**
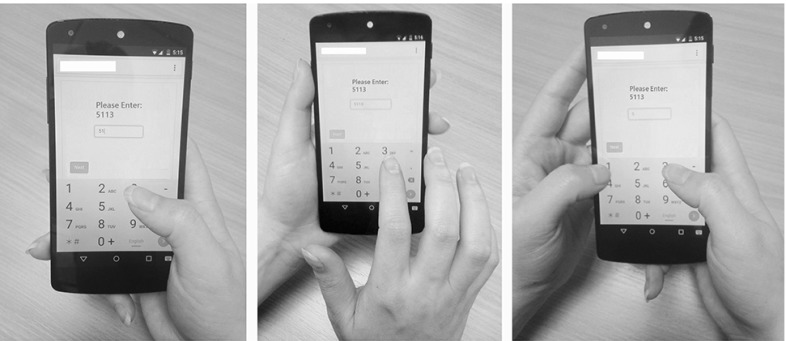
Different input methods used by the users for PIN entrance

We implemented a web page with embedded JavaScript code in order to collect the data from voluntary users. Our code registers two listeners on the window object to have access to orientation and motion data separately. The event handlers defined for these purposes are named *DeviceOrientationEvent* and *DeviceMotionEvent*, respectively. On the client side, we developed a GUI in HTML5 which shows random 4-digit PINs to the users and activates a numpad for them to enter the PINs as shown in Fig. 4. All sensor sequences are sent to the database along with their associated labels which are the digits of the entered PINs. We implemented our server program using Node.js (nodejs.org). Our code sends the orientation and motion sensor data of the mobile device to our NoSQL database using MongoLab (mongolab.com, web-based service for MongoDB). When the event listener fires, it establishes a socket by using Socket.IO (socket.io) between the client and the server and constantly transmits the sensor data to the database. Both Node.js and MongoDB (as a document-oriented database) are known for being capable of supporting data intensive applications in real time.

In the proof-of-concept implementation of the attack, we focus on working with active web pages, which allows us to easily identify the start of a touch action through the JavaScript access to the *onkeydown* event. A similar approach is adopted in other works, e.g. TouchLogger [8] and TapLogger [16]. In an extended attack scenario, a more complex segmentation process would be needed to identify the start and end of a touch action. This could be achieved by measuring the peak amplitudes of a signal, as done in [12].

### Data collection

Following the approach of Aviv et al. [7] and Spreitzer [15], we consider a set of 50 fixed PINs with uniformly distributed digits. We created these PINs in a way that all digits are repeated about the same time (around 20 times). The data collection code is publicly available via GitHub. Technical details of the data collection process and the collected data are publicly available too.^2^

We conducted our user studies using Chrome on an Android device (Nexus 5). The experiments and results are based on the collected data from 10 users, each entering all the 50 4-digit PINs for 5 times. Our voluntary participants were university students and staff and performed the experiments at university offices. We simply explained to them that all they needed was to enter a few PINs shown in a web page.

In relation to the environmental setting for the data collection, we asked the users to remain sitting in a chair while working with the phone. We did not require our users to hold the phone in any particular mode (portrait or landscape) or work with it by using any specific input method (using one or two hands). We let them choose their most comfortable posture for holding the phone and working with it as they do in their usual manner. While watching the users during the experiments, we noticed that all of our users used the phone in the portrait mode by default. Users were either leaning their hands on the desk or freely keeping them in the air. We also observed the following input methods used by the users.

- Holding the phone in one hand and entering the PIN with the thumb of the same hand (Fig. 4 left).
- Holding the phone in one hand and entering the PIN with the fingers of the other hand (Fig. 4 centre).
- Holding the phone with two hands and entering the PIN with the thumbs or fingers of both hands (Fig. 4 right).

In the first two cases, users exchangeably used either their right hands or left hands in order to hold the phone. In order to simulate a real-world data collection environment, we took the phone to each user’s workspace and briefly explained the experiment to them, and let them complete the experiment without our supervision. All users found this way of data collection very easy and could finish the experiments without any difficulties. Our participants were given each an Amazon voucher (worth £10) at the end for their participation.

### Feature extraction

In order to build the feature vector as the input to our classifier algorithm, we consider both time-domain and frequency-domain features. We improve our suggested feature vectors in [4] by adding some more complex features such as the correlation between the measurements. This addition improves the results, as we will discuss in Sect. 4. As discussed before, 12 different sequences obtained from the collected data include orientation (ori), acceleration (acc), acceleration including gravity (accG), and rotation rate (rotR) with three sequences (either *x*, *y* and *z*, or $\alpha$, $\beta$ and $\gamma$) for each sensor measurement. As a pre-processing step and in order to remove the effect of the initial position and orientation of the device, we subtract the initial value in each sequence from subsequent values in the sequence.

We use these pre-processed sequences for feature extraction in time domain directly. In frequency domain, we apply the fast Fourier transform (FFT) on the pre-processed sequences and use the transformed sequences for feature extraction. In order to build our feature vector, first we obtain the maximum, minimum, and average values of each pre-processed and FFT sequences. These statistical measurements give us $3\times 12 = 36$ features in the time domain and the same number of features in the frequency domain. We also consider the total energy of each sequence in both time and frequency domains calculated as the sum of the squared sequence values, i.e., $E=\sum {v_i^2}$ which gives us 24 new features.

The next set of features are in time domain and are based on the correlation between each pair of sequences in different axes. We have 4 different sequences; ori, acc, accG, and rotR, each represented by 3 measurements. Hence, we can calculate 6 different correlation values between the possible pairs; (ori, acc), (ori, accG), (ori, rotR), (acc, accG), (acc, rotR), and (accG, rotR), each presented in a vector with 3 elements. We use the Correlation coefficient function in order to calculate the similarity rate between the mentioned sequences. The correlation coefficient method is commonly used to compare the similarity of the shapes of two signals (e.g. [17]). Given two sequences *A* and *B* and $\mathrm {Cov}(A,B)$ denoting covariance between *A* and *B*, the correlation coefficient is computed as below:

1 $$\begin{aligned} {R_{AB}} = {\mathrm {Cov}(A,B) \over \sqrt{\mathrm {Cov}(A,A) \cdot \mathrm {Cov}(B,B)}} \end{aligned}$$

The correlation coefficient of two vectors measures their linear dependence by using covariance. By adding these new 18 features, our feature vector consists of a total of 114 features.

### Neural network

We apply a supervised machine learning algorithm by using an artificial neural network (ANN) to solve this classification problem. The input of an ANN system could be either raw data, or pre-processed data from the samples. In our case, we have pre-processed our samples by building a feature vector as described before. Therefore, as input, our ANN receives a set of 114 features for each sample. As explained before, we collected 5 samples per each 4-digit PIN from 10 users. While reading the records, we realized that some of the PINs have been entered wrongly by some users. This was expected since each user was required to enter 250 PINs. Since we recorded both expected and entered PINs in our data collection, we could easily identify these PINs and exclude them from our analysis. Overall, out of 2500 records collected from 10 users, 12 of the PINs were entered wrongly. Hence we ended up with 2488 samples for our ANN.

The feature vectors are mapped to specific labels from a finite set: i.e., 50 fixed random 4-digit PINs. We train and validate our algorithm with two different subsets of our collected data, and test the neural network against a separate subset of the data. We train the network with 70% of our data, validate it with 15% of the records, and test it with the remaining 15% of our data set. We use a pattern recognition/classifying network in MATLAB with one hidden layer and 1000 nodes. Pattern recognition/classifying networks normally use a scaled conjugate gradient (SCG) back-propagation algorithm for updating weight and bias values in training. Scaled conjugate gradient is a fast supervised learning algorithm [18].

## Evaluation

In this section, we present the results of our attack on 4-digit PINs in two different forms: multi-users mode and same-user mode. We also train separate ANN systems to learn individual digits of PINs and compare these results with other works.

### Multiple-users mode

The second column of Table 1 shows the accuracy of our ANN trained with the data from all users. In this mode, the results are based on training, validating, and testing our ANN using the collected data from all of our 10 participants. As the table shows, in the first attempt PINlogger.js is able to infer the user’s 4-digit PIN correctly with accuracy of 74.43%, and as expected it gets better in further attempts. By comparison, a random attack can guess a PIN from a set of 50 PINs with the probability of 2% in the first attempt and 6% in three attempts.

**Table 1:** PINlogger.js’s PIN identification rates in different attempts

Attempts	Multiple-users (%)	Same-user (%)
One	74	79
Two	86	93
Three	94	97

### Same-user mode

In order to study the impact of individual training, we trained, validated, and tested the network with the data collected from one user. We refer to this mode of analysis as the same-user mode. We asked our user to enter 50 random PINs, each five times, and repeated the experiment for 10 times (rounds). The reason we have repeated the experiments is that the classifier needs to receive enough samples to be able to train the system. Interestingly, our user used all three different input methods shown in Fig. 4 during the PIN entrance. As expected, our classifier performs better when it is personalized: the accuracy reaches 79.23% in the first attempt and increases to 93.52 and 97.71% in two and three attempts, respectively.

In the same-user mode, convincing the users to provide the attacker with sufficient data for training customized classifiers is not easy, but still possible. Approaches similar to gaming apps such as Math Trainer^3^ could be applied. Math-based CAPTCHAs are possible web-based alternatives. Any other web-based game application which segments the GUI similar to a numerical keypad will do as well. Nonetheless, in this paper we mainly follow the multiple-users approach.

### Identification of PIN digits

One might argue that the attack should be evaluated against the whole 4-digit PIN space. However, we believe that the attack could still be practical when selecting from a limited set of PINs since users do not select their PINs randomly [19]. It has been reported that around 27% of all possible 4-digit PINs belong to a set of 20 PINs,^4^ including straightforward ones like “1111”, “1234”, or “2000”. Nevertheless, we present the results of our analysis of the attack against the entire search space for the two experiment modes discussed above. We considered 10 classes of the entered digits (0–9) from the data we collected on 4-digit PINs used in Sect. 4.1.

In the multiple-users mode, we trained, validated, and tested our system with data from all 10 users. In the same-user mode, we trained personalized classifiers for each user. Unlike the test condition of Sect. 4.2, we did not have to increase the number of rounds of PIN entry here since we had enough samples for each digit per user. In the same-user mode in this section, we used the average of the results of our 10 users. The average identification rates of different digits for three different approaches are presented in Table 2.

**Table 2:** Average digit identification rates in different attempts

Attempts	Multiple-users (%)	Same-user (%)
One	70	79
Two	83	90
Three	92	96

The results in our multiple-users mode indicate that we can infer the digits with a success probability of 70.75, 83.27, and 94.03% in the first, second, and third attempts, respectively. This means that for a 4-digit PIN and based on the obtained sensor data, the attacker can guess the PIN from a set of $3^4 = 81$ possible PINs with a probability of success of $0.9206^4 = 71.82\%$. A random attack, however, can only predict the 4-digit PIN with the probability of 0.81% in 81 attempts. By comparison, PINlogger.js achieves a dramatically higher success rate than a random attacker.

**Table 3:** Comparison of PINlogger.js with related attacks on 4-digit PINs

Features work	Sensor	Access	Training	Identification rate		
				1st try (%)	2nd try (%)	5th try (%)
PIN Skimming [15]	Light	In-app	Same-user	NA	50	65
PIN Skimmer [14]	Cam, Mic	In-app	Same-user	NA	30	50
Keylogging by Mic [12]	Mic, Gyr	In-app	Same-user	94	NA	NA
TapLogger [16]	Acc, Ori	In-app	Same-user	40	75	100
Acc side channel [7]	Acc	In-app	Same-user	18	NA	43
PINlogger.js	Motion, Ori	In-browser	Multiple-users	74	86	98
			Same-user	79	93	99

Using a similar argument, in the same-user mode the success probability of guessing the PIN in 81 attempts is 85.46%. In the same setting, Cai and Chen report a success rate of 65% using accelerometer and gyroscope data [20] and Simon and Anderson’s [14] PIN Skimmer only achieves a 12% success rate in 81 attempts using camera and microphone. Our results in digit recognition in this paper are also better than what is achieved in TouchSignatures [4]. In summary, PINlogger.js performs better than all sensor-based digit-identifier attacks in the literature.

### Comparison with related work

Obtaining sensitive information about users such as PINs based on mobile sensors has been actively explored by researchers in the field [21, 22]. In particular, there is a number of research which uses mobile sensors through a malicious app running in the background to extract PINs entered on the soft keyboard of the mobile device. For example, GyroPhone, by Michalevsky et al. [10], shows that gyroscope data are sufficient to identify the speaker and even parse speech to some extent. Other examples include Accessory [13] by Owusu et al. and Tapprints by Miluzzo et al. [11]. They infer passwords on full alphabetical soft keyboards based on accelerometer measurements. Touchlogger [8] is another example by Cai and Chen [20] which shows the possibility of distinguishing user’s input on a mobile numpad by using accelerometer and gyroscope. The same authors demonstrate a similar attack in [9] on both numerical and full keyboards. The only work which relies on in-browser access to sensors to attack a numpad is our previous work, TouchSignatures [4]. All of these works, however, aim for the individual digits or characters of a keyboard, rather than the entire PIN or password.

Another category of works directly targets user PINs. For example, PIN skimmer by Simon and Anderson [14] is an attack on a user’s numpad and PINs using the camera and microphone on the smartphone. Spreitzer suggests another PIN Skimming attack [15] and steals a user’s PIN based on the measurements from the smartphone’s ambient light sensor. Narain et al. introduce another attack [12] on smartphone numerical and alphabetical keyboards and the user’s PINs and credit card numbers by using the smartphone microphone. TapLogger by Xu et al. [16] is another attack on the smartphone numpad which outputs the pressed digits and PINs based on accelerometer and orientation sensor data. Similarly, Aviv et al. introduce an accelerometer-based side channel attack on the user’s PINs and patterns in [7]. We choose to compare PINlogger.js with the works in this category since they have the same goal of revealing the user’s PINs. Table 3 presents the results of our comparison.

As shown in Table 3, PINlogger.js is the only attack on PINs which acquires the sensor data via JavaScript code. In-browser JavaScript-based attacks impose even more security threats to users since unlike in-app attacks, they do not require any app installation and user permission to work. Moreover, the attacker does not need to develop different apps for different platforms such as Android, iOS, and Windows. Once the attacker develops the JavaScript code, it can be deployed to attack all mobile devices regardless of the platform. Moreover, Touchlogger.js is the only works which present the results of the attack for multiple-users modes. By contrast, the results from other works are mainly based on training the classifiers for individual users. In other words, they assume the attacker is able to collect input training data from the victim user before launching the PIN attack. We do not have such an assumption as the training data are obtained from all users in the experiment. In terms of accuracy, with the exception of [12], PINlogger.js generally outperforms other works with an identification rate of 74% in the first attempt. This is a significant success rate (despite that the sampling rate in-browser is much lower than that available in-app) and confirms that the described attack imposes a serious threat to the users’ security and privacy.

## Why does this vulnerability exist?

Although reports of side channel attacks based on the in-browser access to mobile sensors via JavaScript are relatively recent, similar attacks via in-app access to mobile sensors have been known for years. Yet the problem has not been fixed. Here, we discuss the reasons why such a vulnerability has remained unfixed for a long time.

**Table 4:** Motion sensors supported by Android and their corresponding W3C definitions

Android motion sensors	Description	Unit	W3C def.
Accelerometer	Acceleration force along 3 axes	m/s$^2$	Acceleration with gravity
Gravity	Force of gravity along 3 axes	m/s$^2$	NA
Gyroscope	Rate of rotation around 3 axes	rad/s	Rotation rate
Uncalibrated gyroscope	Rate of rotation (no drift compensation), and	rad/s	NA
	Estimated drift around 3 axes	rad/s	NA
Linear accelerometer	Acceleration force excluding gravity along 3 axes	m/s$^2$	Acceleration
Rotation vector	Rotation vector component along 3 axes	Unitless	NA
Step counter	Number of user’s steps since last reboot	Steps	NA

**Table 5:** Position sensors supported by Android and their corresponding W3C definitions

Android position sensors	Description	Unit	W3C def.
Game rotation vector	Rotation vector component along 3 axes	Unitless	NA
Geomagnetic rotation vector	Rotation vector component along 3 axes	Unitless	NA
Geomagnetic magnetic field	Geomagnetic field strength along 3 axes	$\upmu$T	NA
Uncalibrated magnetic field	Geomagnetic field strength (no hard iron calibration)	$\upmu$T	NA
	And iron bias estimation along 3 axes	$\upmu$T	NA
Orientation	Angles around 3 axes	Degrees	Orientation
Proximity	Distance from object	cm	NA

Orientation sensor was deprecated in Android 2.2 (API Level 8)

### Unmanaged sensors

In an attempt to explain multiple sensor-related in-app vulnerabilities, Xu et al. [16] argue that “the fundamental problem is that sensing is unmanaged on existing smartphone platforms”. There are multiple in-app side-channel attacks that support this argument, as we discussed in the previous section. Our work shows that the problem of in-app access to “unmanaged sensors” is now spreading to in-browser access. Here we present the “unmanaged” motion and orientation sensor case which shows how the technical mismanagement of these sensors causes serious user privacy consequences when it comes to unregulated access to such sensors via JavaScript.

*W3C vs. Android* According to W3C specifications [6], the motion and orientation sensor streams are not raw sensor data, but rather high-level data which are agnostic to the underlying source of information. Common sources of information include gyroscopes, compasses, and accelerometers. In Tables 4 and 5, we present raw (low-level) and synthesized (high-level) motion sensors supported by Android [1] along with their descriptions and units, as well as their corresponding W3C definitions [6].

As it can be seen from the tables, different terminologies have been used for describing the same measurements in-app and in-browser. For example, while in-app access uses the raw sensor terminology, i.e., accelerometer, gyroscope, magnetic field, the in-browser access uses synthesized sensor terminology, i.e., motion and orientation [6]. This creates confusion for users (as we will explain later) and developers (as we experienced it ourselves). One of the W3C’s specifications on mobile sensors, “Generic Sensor API” [23], dedicates a few sections to the issue of naming sensors, and low-level and high-level sensors. It discusses how the terminology for in-browser access has been high-level so far. It also mentions that the low-level use cases are increasingly popular among the developers. As stated in this specification: “The distinction between high-level and low-level sensor types is somewhat arbitrary and the line between the two is often blurred”. And “Because the distinction is somewhat blurry, extensions to this specification are encouraged to provide domain-specific definitions of high-level and low-level sensors for the given sensor types they are targeting”. We believe that due to the rapid increase in mobile sensors, it is necessary to come up with a consistent approach.

### Unknown sensors

We believe another contributing factor is that users seem to be less familiar with the relatively newer (and less advertised) sensors such as motion and orientation, as opposed to their immediate familiarity with well-established sensors such as camera and GPS. For example, a user has asked this question on a mobile forum: “$\ldots$ What benefits do having a gyroscope, accelerometer, proximity sensor, digital compass, and barometer offer the user? I understand it has to do with the phone orientation but am unclear in their benefits. Any explanation would be great! Thanks!”.^5^

We design and conduct user studies in this work in order to investigate to what extent are these sensors and their risks known to the users.

*List of mobile sensors* We prepared a list of different mobile sensors by inspecting the official websites of the latest iOS and Android products, and the specifications that W3C and Android provide for developers. We also added some extra sensors as common sensing mobile hardware which are not covered before.

- iPhone 6^6^: Touch ID, Barometer, Three-axis gyro, Accelerometer, Proximity sensor, Ambient light sensor.
- Nexus 6P^7^: Fingerprint sensor, Accelerometer, Gyroscope, Barometer, Proximity sensor, Ambient light sensor, Hall sensor, Android Sensor hub.
- Android [1]: Accelerometer, Ambient temperature, Gravity (software or hardware), Gyroscope, Light, Linear Acceleration (software or hardware), Magnetic Field, Orientation (software), Pressure, Proximity, Relative humidity, Rotation vector (software or hardware), Temperature.
- W3C^8^ [6]: Device orientation (software), Device motion (software), Ambient light, Proximity, Ambient temperature, Humidity, Atmospheric Pressure.
- Extra sensors (common sensing hardware): Wireless technologies (WiFi, Bluetooth, NFC), Camera, Microphone, Touch screen, GPS.

Unless specified otherwise, all the listed sensors are hardware sensors. We added the last category of the sensors to this list since they indeed sense the device’s surrounding although in different ways. However, they are neither counted as sensors in mobile product descriptions, nor in technical specifications. These sensors are often categorized as OS resources [24], and hence, different security policies apply to them.

### User study

In this section, we aimed to observe the amount of knowledge that mobile users have about mobile sensors. We prepared a list of sensors based on what we explained above and asked volunteer participants to rate the level of their familiarity with each sensor. All of our experiments and user studies were approved by Newcastle University’s ethical committee.

#### Participants

We recruited 60 participants to take part in this study via different means including mailing lists, social networking, vocational networks, and distributing flyers in different places such as different schools in the university, colleges, local shops, churches, and mosques. A sample of our call for participation and participants’ demographics are available in “Appendix 1”.

Among our participants, 28 self-identified themselves as male and 32 as female, from 18 to 67 years old, with a median age of 33.85. None of the participants were studying or working in the field of mobile sensor security. Our university participants were from multiple degree programs and levels, and the remaining participants worked in a different range of fields. Moreover, our participants owned a wide range of mobile devices and had been using a smartphone/tablet for 5.6 years on average. Our participants were from different countries, and all could speak English. We interviewed our participants at a university office and gave each an Amazon voucher (worth £10) at the end for their participation. Details of the interview template can be found in “Appendix 2”.

#### Study approach

For a list of 25 different sensors, we used a five-point scale self-rated familiarity questionnaire as used in [25]: “I’ve never heard of this”, “I’ve heard of this, but I don’t know what this is”, “I know what this is, but I don’t know how this works”, “I know generally how this works”, and “I know very well how this works”. The list of sensors was randomly ordered for each user to minimize bias. In addition, we needed to observe the experiments to make sure users were answering the questions based on their own knowledge in order to avoid the effect of processed answers. Full descriptions of all studies are provided in “Appendix 2”.

**Fig. 5:**
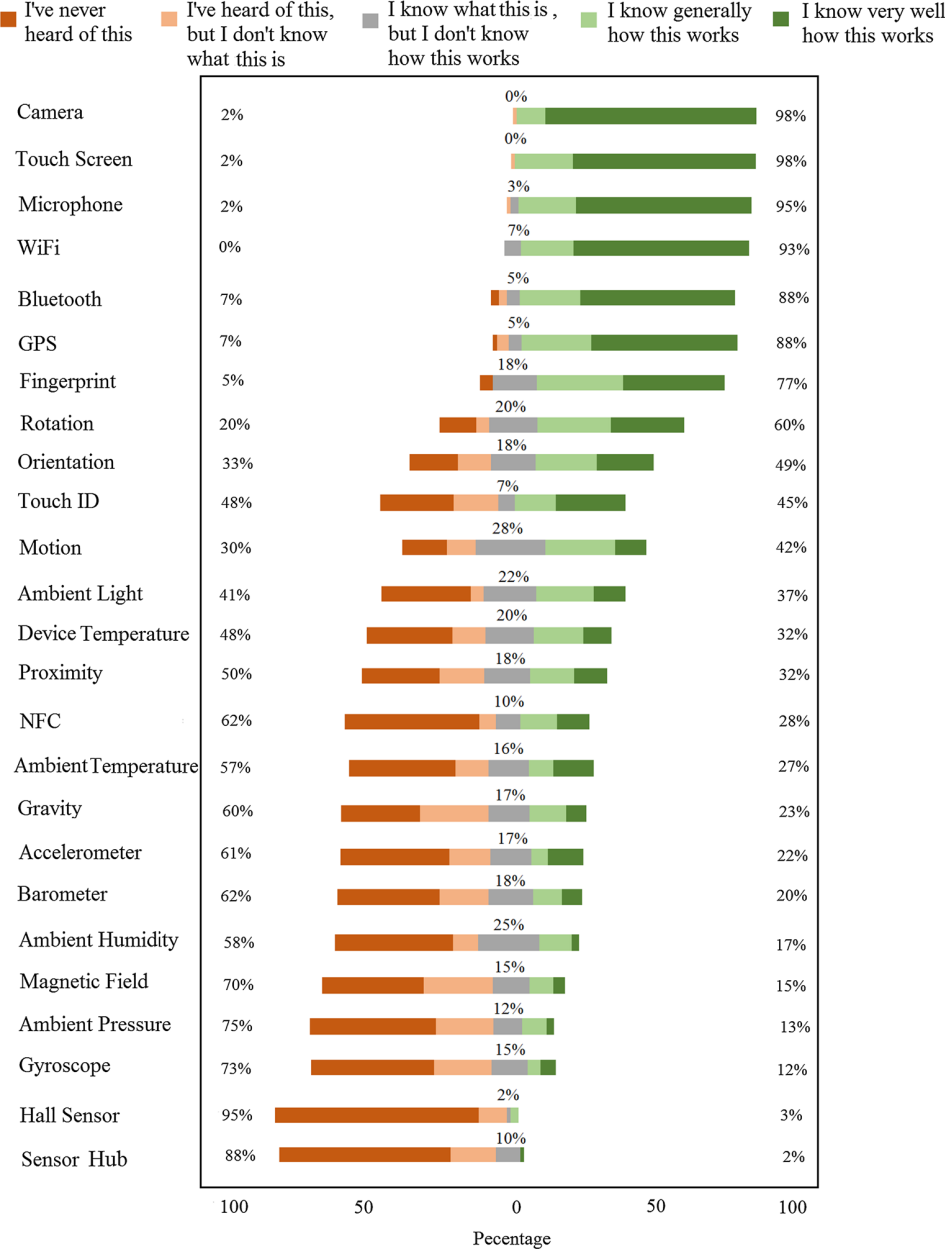
Level of self-declared knowledge about different mobile sensors

#### Findings

Figure 5 summarizes the results of this study. This figure shows the level of self-declared knowledge about different mobile sensors. The question was: “To what extent do you know each sensor on a mobile device?”. Sensors are ordered based on the aggregate percentage of participants declaring they know generally or very well how each sensor works. This aggregate percentage is shown on the right-hand side. In the case of equal aggregate percentage, the sensor with a bigger share on being known very well by the participants is shown earlier. Our participants were generally surprised to hear about some sensors and impressed by the variety. As one may expect, newer sensors tend to be less known to the users in comparison with older ones. In particular, our participants were generally not familiar with ambient sensors. Although some of our participants knew the ambient sensors in other contexts (e.g. thermostats used at home), they could not recognize them in the context of a mobile device.

Low-level hardware sensors such as accelerometer and gyroscope seem to be less known to the users in comparison with high-level software ones such as motion, orientation, and rotation. We suspect that this is partly due to the fact that the high-level sensors are named after their functionalities and can be more immediately related to user activities.

We also noticed that a few of the participants knew some of the low-level sensors by name but they could not link them to their functionality. For example, one of our participants who knew almost all of the listed sensors (except hall sensor and sensor hub) stated that: “When I want to buy a mobile [phone], I do a lot of search, that is why I have heard of all of these sensors. But, I know that I do not use them (like accelerometer and gyroscope)”.

On the other hand, as the functionalities of mobile devices grow, vendors quite naturally turn to promote the software capabilities of their products, instead of introducing the hardware. For example, many mobile devices are recognized for their gesture recognition features by the users; however, the same users might not know how these devices provide such a feature. For instance, one of the participants commented on a feature on her smartphone called “Smart Stay”^9^ as follows: “I have another sensor on my phone: Smart Stay. I know how it works, but I don’t know which sensors it uses”.

## User studies on risk perception of mobile sensors

In this section, we study the participants’ risk perception of mobile sensors. There have been several studies on risk perception addressing different aspects of mobile technology. Some works discuss the risks that users perceive on smartphone authentication methods such as PINs and patterns [26], TouchID and Android face unlock [27], and implicit authentication [28]. Other works focus on the privacy risks of certain sensors such as GPS [29]. Raji et al. [30] show users’ concerns (on disclosure of selected behaviours and contexts) about a specific sensor-enabled device called AutoSense^10^. To the best of our knowledge, the research presented in this paper is the first that studies the user risk perception for a comprehensive list of mobile sensors (25 in total). We limit our study to the level of perceived risks users associate with their PINs being discovered by each sensor. The reasons we chose PINs are that first, finding one’s PIN is a clear and intuitive security risk, and second, we can put the perceived risk levels in context with respect to the actual risk levels for a number of sensors as described in Table 3.

### Methodology

For this study, we divide our 60 participants into two groups and studied the two group separately using two different approaches: within-subject and between-subject. In the within-subject study, we interviewed 30 participants for all parts of the study. In contrast, in the between-subject study, we interviewed a new group of 30 participants, and we later compared the results with the previous group. By these two approaches, we aim to measure differences (after informing users on descriptions of sensors) within a participant and between participants, respectively.

#### Within-subject study

In this approach, we asked 30 participants to rate the level of risk they perceive for each sensor in regard to revealing their PINs in two phases. In phase one, we gave the same sensor list (randomized for each user). We described a specific scenario in which a game app which has access to all these sensors is open in the background and the user is working on his online banking app, entering a PIN. We used a self-rated questionnaire with five-point scale answers following the same terminology as used in [30]: “Not concerned”, “A little concerned”, “Moderately concerned”, “Concerned”, and “Extremely concerned”. During this phase, we asked the users to rely on the information that they already had about each sensor (see “Appendix 2” for details).

In the second phase, first we provided the participants with a short description of each sensor and let them know that they can ask further questions until they feel confident that they understand the functionality of all sensors. Participants could use a dictionary on their device to look at the words that were less familiar to them. Afterwards, we asked the participants to fill in another copy of the same questionnaire on risk perceptions (details in “Appendix 2”). Participants could keep the sensor description paper during this phase to refer to it in the case they forgot the description of certain sensors.

#### Between-subject study

In this study, first we gave the description of the sensors to our second group of 30 participants, and similar to previous study, we gave them enough time to familiarize themselves with the sensors and to ask as many questions as they wanted until they felt confident about each sensor. Then, we presented the participants with the questionnaire on risk perceptions (details in “Appendix 2”). Similar to our previous study, participants could keep the sensor description paper while filling in this questionnaire.

**Fig. 6:**
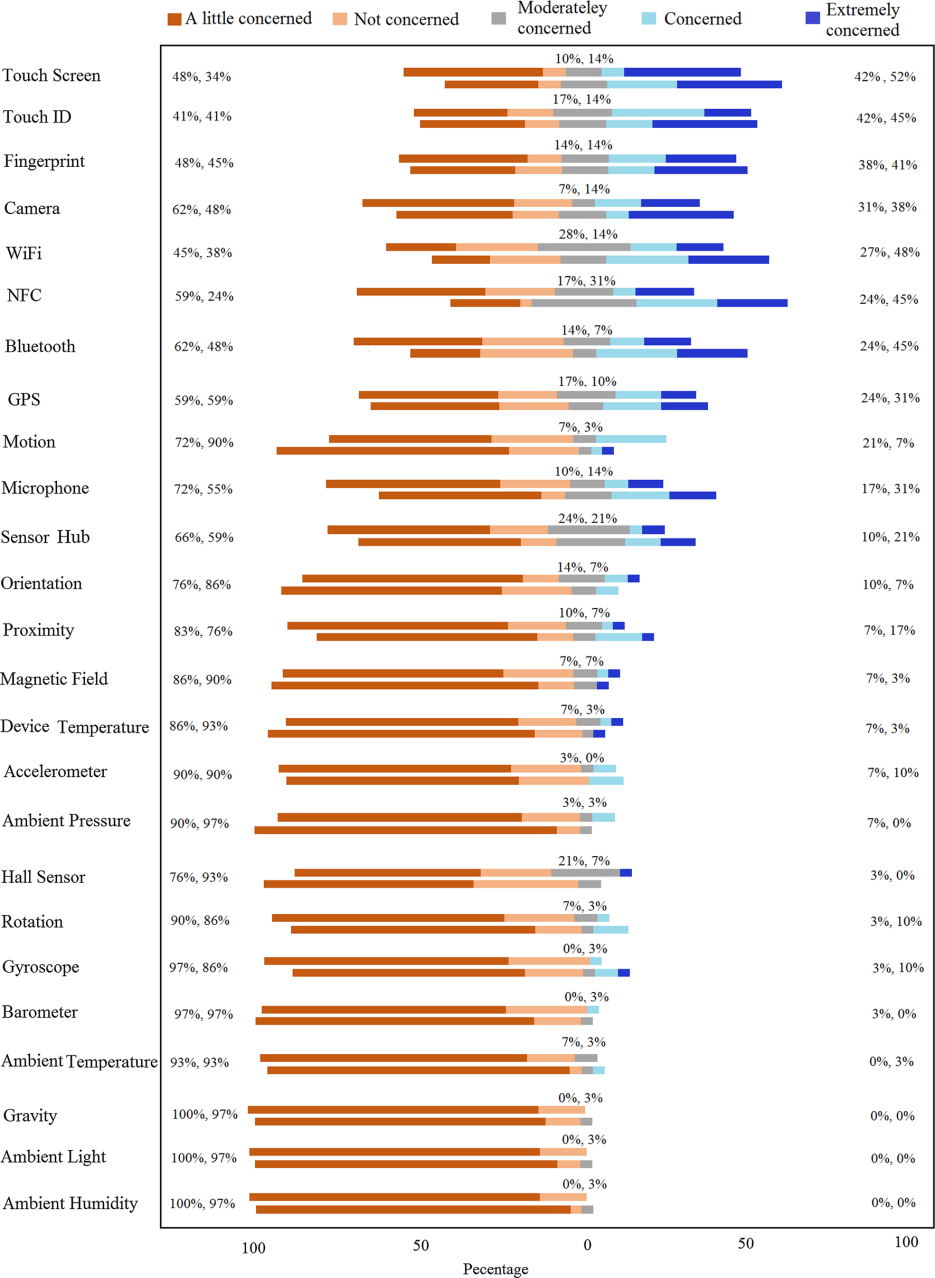
Users’ perceived risk for different mobile sensors for within-subject approach

**Fig. 7:**
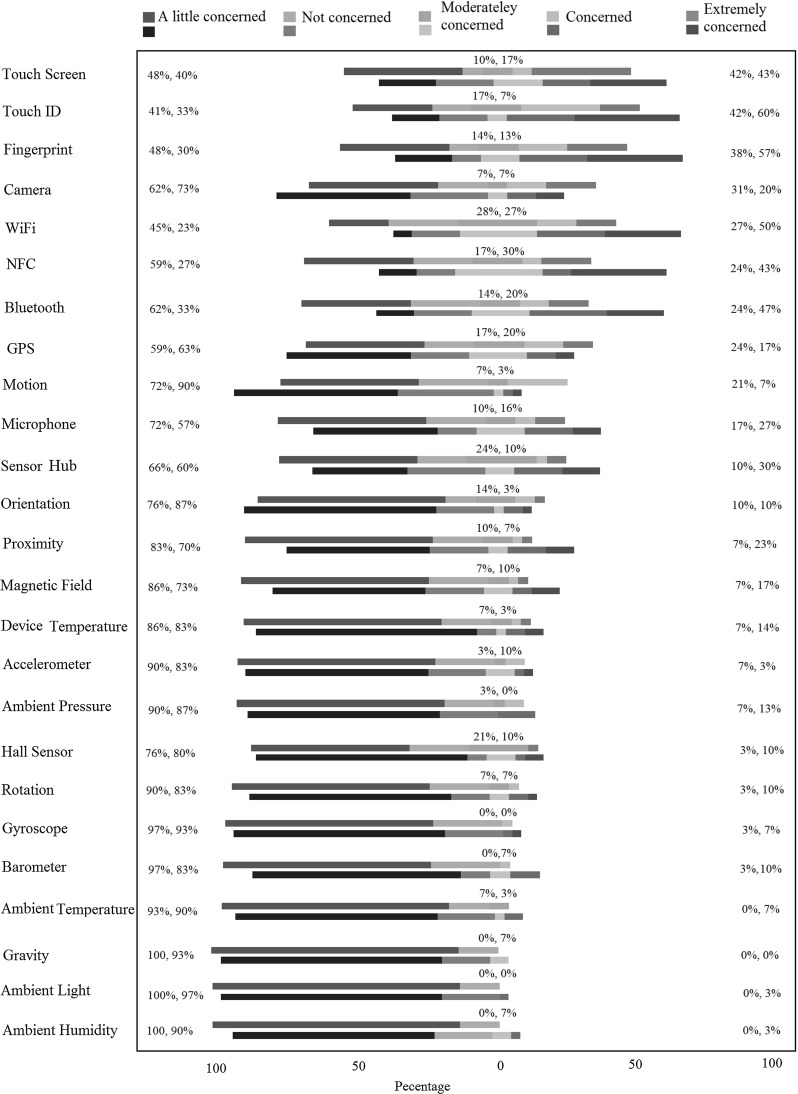
Users’ perceived risk for different mobile sensors for between-subject approach

### Intuitive risk perception

The results of our within-subject study are presented in Fig. 6. These results present the users’ perceived risk for different mobile sensors for the same group of users before (top bars) and after (bottom bars) being presented with descriptions of sensors. The results of our between-subject study are presented in Fig. 7. Note that this figure represents the risk perception of group one of our participants before knowing the sensors descriptions, and group two of participants after knowing the sensors descriptions. For both figures, the question was: “To what extent are you concerned about each sensor’s risk to your PIN?”, sensors are ordered based on the aggregate percentage of participants declaring they are either concerned or extremely concerned about each sensor before seeing the descriptions. This aggregate percentage is the first value presented on the right-hand side. In the case of equal aggregate percentage, the sensor with a bigger share on being perceived extremely concerned by the participants is shown earlier.

We make the following observations from the results of the experiment.

*Touch Screen* Although our participants rated touch screen as one of the most risky sensors in relation to a PIN discovery scenario, still about half of our participants were either moderately concerned, a little concerned, or not concerned at all. Through our conversations with the users, we received some interesting comments, e.g. “Why any of these sensors should be dangerous on an app while I have officially installed it from a legal place such as Google Play?”, and “As long as the app with these sensors is in the background, I have no concern at all”. It seems that a more general risk model in relation to mobile devices is affecting the users’ perception in regard to the presented PIN discovery threat. This fact can be a topic of research on its own and is out of the scope of this paper.

*Communicational Sensors* One category of the sensors which users are relatively more concerned about includes WiFi, Bluetooth, and NFC. For example, one of the participants commented that: “I am not concerned with physical [motion, orientation, accelerometer, etc.]/ environmental [light, pressure, etc.] sensors, but network ones. Hackers might be able to transfer my information and PIN”. These sensors appearing more risky to the users are understandable since we asked them to what extent they were concerned about *each sensor* in regard to the PIN discovery.

*Identity-Related Sensors* Another category which has been rated more risky than others contains those sensors which can capture something related to the user’s identity, i.e. fingerprint, TouchID, GPS, camera, and microphone. Despite that we described a PIN-related scenario, our participants were still concerned about these sensors. This was also pointed out by a few participants through the comments. For example, a user stated: “$\ldots$, however, GPS might reveal the location along with the user input PIN that has a risk to reveal who (and where) that PIN belongs to. Also the fingerprint/TouchID might recognize and record the biometrics with the user’s PIN”. Some of these sensors such as GPS, fingerprint, and TouchID, however, cannot cause the disclosure of PINs on their own. Hence, the concern does not entirely match the actual risk. Similar to the discussion on touch screen, we believe that a more general risk model on mobile technology influences the users to perceive risk on specific threats such as the one we presented to them.

*Environmental Sensors* The level of concern on ambient sensors (humidity, light, pressure, and temperature) is generally low and stays low after the users are provided with the description of the sensors (see Fig. 6). In many cases, our users expressed that they were concerned about these sensors simply because they did not know them: “[now that I know these sensors,] I am quite certain that movement/environmental sensors would not affect the security of personal id/passwords, etc.”. In fact, researchers have reported that it is possible to infer the user’s PIN using the ambient light sensor data [15], although, to our knowledge, exploits of other environmental sensors have not been reported in the literature.

*Movement Sensors* On the sensors related to the movement and the position of the phone (accelerometer, gyroscope, motion, orientation, and rotation), the users display varying levels of the risk perceptions. In some cases, they are slightly more concerned, but in others they are less concerned once they know the functionality. Some of our users stated that since they did not know these sensors, they were not concerned at all, but others were more concerned when they were faced with new sensors. Overall, knowing or not knowing these sensors has not affected the perceived risk level significantly, and they were rated generally low in both cases.

*Motion and Orientation Sensors* The sensors which we used in our attack, namely orientation, rotation, and motion, have not been generally scored high for their risk in revealing PINs. Users do not seem to be able to relate the risk of these sensors to the disclosure of their PINs, despite that they seem to have an average general understanding about how they work. On hardware sensors such as accelerometer and gyroscope, the risk perception seems to be even lower. A few comments include: “In my everyday life, I don’t even think about these [movement] sensors and their security. There is nothing on the news about their risk”, and “I have never been thinking about these [movement] sensors and I have not heard about their risk”. On the other hand, some of the participants expressed more concerns for sensors that they were familiar with, as one wrote, “You always hear about privacy stuff for example on Facebook when you put your location or pictures”. Similarly, it seems that having a previous risk model is a factor that might explain the correlation between the user’s knowledge and their perceived risk.

## Discussions

### General knowledge versus risk perception

Figures 5 and 6 suggest that there may be a correlation between the relative level of knowledge users have about sensors and the relative level of risk they perceive from them. We confirm our observation of correlation using Spearman’s rank-order correlation measure. As shown in Table 6, we present the Spearman’s correlation between the comparative knowledge and the perceived risk about different sensors for different participants’ data set: group one before being presented with the sensor descriptions, group one after sensor description, group two after sensor descriptions, and finally groups one and two after being presented with the sensor descriptions.

For each participants’ data set, the sensors are separately ranked based on the level that the users are familiar with them, similar to Fig. 5. Accordingly, the levels of concern are ranked too. The Spearman’s correlation equation has been applied on these ranks for each group separately.

For example, the Spearman’s correlation between the comparative knowledge (median: “I know what this is, but I don’t know how this works”, IQR^11^: “I’ve never heard of this”–“I know very well how this works”) and the perceived risk about different sensors for group one (median: “Not concerned”, IQR: “Not concerned”–“A little concerned”) before knowing the sensor descriptions is $r = 0.61$ ($p<0.05$).

As it can be seen, these results support that the more the users know about these sensors, the more concern they express about the risk of the sensors revealing PINs. We acknowledge that other methods of ranking the results, e.g. using median, produce slightly different final rankings. However, given the high confidence level of the above test, we expect the correlation to be supported if other methods of ranking are used.

**Table 6:** Spearman’s correlation between the comparative knowledge and the perceived risk about different sensors

Participants’ data set	Status	Spearman’s correlation
Group 1	Before sensor desc.	0.61
Group 1	After sensor desc.	0.61
Group 2	After sensor desc.	0.48
Groups 1 and 2	After sensor desc.	0.58

Assuming that customer demand drives better security designs, the above correlation may explain why sensors that are newer to the market have not been considered as OS resources and consequently have not been subject to similar strict access control policies.

### Perceived risk versus the actual risk

We are specifically interested in the users’ relative risk perception of sensors in revealing their PINs in comparison with the actual relative risk level of these sensors. We list the results reported in the literature in Table 3 for the following sensors: light, camera, microphone, gyroscope, motion, and orientation. Figure 6 shows that users generally have expressed more concern about sensors such as camera and microphone than accelerometer, gyroscope, orientation, and motion. This does not match the actual risk levels since the latter sensors allow PIN recovery with higher accuracy as we have shown in Sect. 4. When asked after filling the questionnaire, most participants could not come up with realistic attack scenarios using camera and microphone. For microphone, some users thought they might say the PIN out loud. For camera, a few of our participants thought face recognition might be used to recover the PIN; hence, they rated camera’s risk to their PINs high. One user thought the camera might capture the reflection of the entered PIN in her glasses.

Among our participants, one mentioned but described doubt about motion, orientation, accelerometer, and gyroscope being able to record the shakes of the mobile phone while entering a PIN after they saw the sensor descriptions: “I feel those positional sensors might be able to reveal something about my activities, for example if I open my banking app or enter my PIN. But it is extremely hard for different users, and when working with different hands and positions”. This participant expressed only “a little concern” about them, stating that: “$\ldots$, and by little concern, I mean extremely little concern”. One of our participants was completely familiar with these attacks and in fact had read some related papers. This user was “extremely concerned”. Other users who rated these sensors risky in general said they were generally concerned about different sensors. One commented: “I can not think of any particular situation in which these sensors can steal my PIN, but the hackers can do everything these days”.

### Possible solutions

In this section, we discuss the current academic and industrial countermeasures to mitigate sensor-based attacks.

#### Academic approach

Different solutions to address the in-app access attacks have been suggested in the literature, e.g. restricting the sensor to one app, reducing the sampling rate, temporal pause of the sensor on sensitive entries such as keyboard, rearranging keyboard for password entrance, asking for explicit permission from the user, ranking apps based on their similarities to malware, and obfuscating anomalies in sensor data [7, 10–16, 31, 32]. However, after many years of research on showing the serious security risks of sensors such as accelerometer and gyroscope, none of the major mobile platforms have revised their in-app access policy.

We believe that the risks of unmanaged sensors on mobile phones, specially through JavaScript code, are not known very well yet. More specifically, many OS-/app-level solutions such as asking for permissions at the installation time or malware detection approaches would not work in the context of a web attack. In our previous work [4], we suggested to apply the same security policies as those for camera, microphone, and GPS for the motion and orientation sensors. Our suggestion was to set a multi-layer access control system on the OS and browser levels. However, the usability and effectiveness of this solution are arguable. First, asking too many permissions from the user for different sensors might not be usable. Furthermore, for some basic use cases such as gesture recognition to clear a web form, or adjusting the screen from portrait to landscape, it might not make sense to ask for user permission for every website. Second, with the increase in the number of sensors accessible through mobile browsers, this approach might not be effective due to the classic problem of sidestepping the security procedure by users when it is too much of a burden [33]. As stated by one of our participants: “I don’t mind these sensors being risky anyway. I don’t even review the permission list. I have no other choice to be able to use the app”. Moreover, as we have shown in Sect. 5, users generally do not understand the implications of these sensors on discovering their PINs, for example, even though they know how these sensors work. Hence, such an approach might not be effective in practice.

#### Industrial approach

**W3C Device Orientation Event Specification.** There is no Security and Privacy section in the latest official W3C Working Draft Document on Device Orientation Event [6]. However, at the time of writing this paper, a new version of the W3C specification is being drafted, which includes a new section on security and privacy issues related to mobile sensors,^12^ as suggested by us in [4]. The authors working on the revision of the W3C specification point out the problem of fingerprinting mobile devices [31], and touch action recovery [4] through these sensors, and suggest the following mitigations:

- “Do not fire events when the page where they were registered on is not visible or has been backgrounded.”
- “Fire events only on the top-level browsing context or same-origin nested iframes.”
- “Limit the frequency of events (typically 60 Hz seems to be sufficient).”

We believe that these measures may be too restrictive in blocking useful functionalities. For example, imagine a user consciously running a web program in the browser to monitor his daily physical activities such as walking and running. This program needs to continue to have access to the motion and orientation sensor data when the user is working on another tab or minimizes the browser. One might argue that such a program should be available as an app instead; hence, the use case is not valid. However, it is expected that the boundary between installed apps and embedded JavaScript programs in the browser will gradually diminish [34].

*Mobile browsers* As we showed in [4], browsers and mobile operating systems behave differently on providing access to sensors. Some allow access only on the active webpage and any embedded iframes (although with different origins), some allow access to other tabs, when browser is minimized, or even when the phone is locked. Hence, there is not a consistent approach across all browsers and mobile platforms. Reducing the frequency rate has been applied to all well-known browsers at the moment [4]. For instance, Chrome reduced the sensor readings from 200 to 60 Hz due to security concerns.^13^ However, our attack shows that security risks are still present even at lower frequencies. iOS and Android limit the maximum frequency rate of some sensors such as Gyroscope to 100 and 200 Hz, respectively. It is expected that these frequencies will increase on mobile OSs in the near future and in-browser access is no exception. In fact, current mobile gyroscopes support much higher sampling frequencies, e.g. up to 800 Hz by STMicroelectronics (on Apple products) and up to 8000 Hz by InvenSense (on the Google Nexus range) [10]. With higher frequencies available, attacks such as ours can perform better in the future if adequate security countermeasures are not applied.

Following our report of the issue to Mozilla, starting from version 46 (released in April 2016), Firefox restricts JavaScript access to motion and orientation sensors to only top-level documents and same-origin iframes.^14^ In the latest Apple Security Updates for iOS 9.3 (released in March 2016), Safari took a similar countermeasure by “suspending the availability of this [motion and orientation] data when the web view is hidden”.^15^ However, we believe the implemented countermeasures should only serve as a temporary fix rather than the ultimate solution. In particular, we are concerned that it has the drawback of prohibiting potentially useful web applications in the future. For example, a web page running a fitness program has a legitimate reason to access the motion sensors even when the web page view is hidden. However, this is no longer possible in the new versions of Firefox and Safari. Our concern is confirmed by members in the Google Chromium team,^16^ who also believe that the issue remains unresolved.

### Biometric sensors

As we explained in Sect. 5.2, there exist around 25 different sensors on mobile platforms. They include communicational sensors such as WiFi, environmental sensors such as ambient light, movement sensors such as motion and orientation, and biometric sensors such as Fingerprint. Here we specifically discuss biometric sensors since they are highly related to the individuals’ identity.

After decades of working on password, it seems that people still cannot remember strong passwords. Biometrics have been offered to users as an effective authentication mechanism. Examples include TouchID and Fingerprint sensors on iOS and Android devices, respectively. But the biometric-based authentication is not limited to mobile devices only. For example, when paying with iPhone contactlessly, you need to rest your finger on TouchID and hold your iPhone in close proximity to the contactless reader until the task is finished. Furthermore, since many banks have already moved their services to mobile platforms, they benefit from the biometrics sensors available on mobile devices, say for implementing 2-factor authentication. As an example, in addition to user name and passwords, HSBC authenticates their customers through TouchID^17^ and voice ID.^18^ Another example is *Smile to Pay* facial recognition app^19^ where deep learning is applied to overcome the difficulty of face authentication when the face photograph is not in the normal form. Recently Yahoo has also introduced its ear-based smartphone identification system.^20^

On the other hand, our findings show that mobile users are relatively concerned with identity-related or biometric sensors. However, we discussed that these sensors are not necessarily the most risky ones to PINs in practice. As we mentioned earlier, we believe that this might be the influence of a more general risk model that the users have on mobile technology. We believe that this is an important research topic and requires further studies.

### Limitations

We consider this work a pilot study that explores user risk perception on a comprehensive list of mobile sensors. We envisage the following future work to address these limitations and expand this work:

- *More Participants* We performed our user studies on a set of users who were recruited from a wide range of backgrounds. Yet the number of the participants is limited. A larger set of participants will improve the confidence in the results. With a large and diverse set of participants, we can also study the effect of demographic factors on perceived risk.
- *Other Risks* We studied the perceived risk on PINs as a serious and immediate risk to users’ security. The study can be expanded by studying users’ risk perception on other issues such as attackers discovering phone call timing, physical activities, or shopping habits.
- *Other Types of Access* When interviewing our participants, we presented them with a scenario involving a game app which is installed on their smartphone. This only covers the in-app access to sensors. However, people might express different risk levels for other types of access, e.g. in-browser access. This needs further investigation.
- *Issues with Training Users* We decided to provide our participants with a short description of each sensor’s functionality (details in “Appendix 2”, part 3). Furthermore, the participants were given the chance to ask as many questions as they wanted to fully understand the functionality of each sensor. This might not be the most effective way to inform users about sensors since some descriptions might seem too technical (and hence not fully understandable) to some users. How to inform users in an effective way is a complex topic of research which can be explored in the future.

## Conclusion

In this paper, we introduced PINlogger.js, a web-based program which reveals users’ PINs by recording the mobile device’s orientation and motion sensor data through JavaScript code. Access to mobile sensor data via JavaScript is limited to only a few sensors at the moment. This will probably expand in the future, specially with the rapid development of sensor-enabled devices in the Internet of things (IoT).

We also showed that users do not generally perceive a high risk about such sensors being able to steal their PINs. Furthermore, we showed that people are not even generally knowledgeable about these sensors on mobile devices. Accordingly, we discussed the complexity of designing a usable and secure solution to prevent the proposed attacks. Hence, designing a general mechanism for secure and usable sensor data management remains a crucial open problem for future research.

Many of the suggested academic solutions either have not been applied by the industry as a practical solution, or have failed. Given the results in our user studies, designing a practical solution for this problem does not seem to be straightforward. A combination of different approaches might help researchers devise a usable and secure solution. Having control on granting access *before* opening a website and *during* working with it, in combination with a smart notification feature in the browser would probably achieve a balance between security and usability. Users should also have control on reviewing, updating and deleting these data, if stored by the website or shared with a third party *afterwards*. Solutions such as Taintroid [35], a tracking app for monitoring sources of sensitive data on a mobile which has been applied for GPS in [29], could be helpful. After all, it seems that an extensive study is required towards designing a permission framework which is usable and secure at the same time. Such research is a very important usable security and privacy topic to be explored further in the future.

## Figures and Tables

**Fig. 8:**
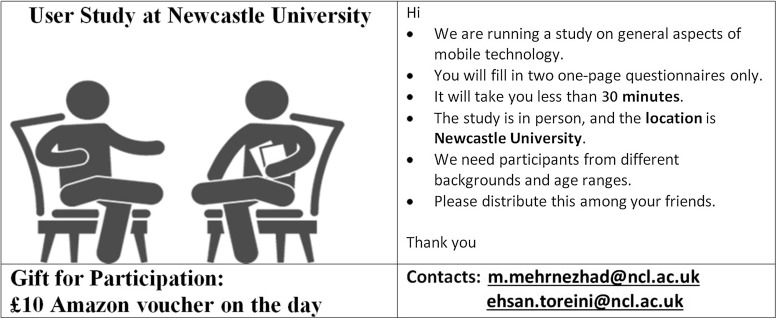
Sample of flyer distributed for participant recruitment

## Appendix 1: Call for participation flyer and participant demographics

In this section, we present the participants demographics in details and the flyers that we used for call for participation of our user studies (Fig. 8; Table 7).

**Table 7:** Participants’ self-reported demographics in the two studies, (*y*) indicates the years of owning a smartphone

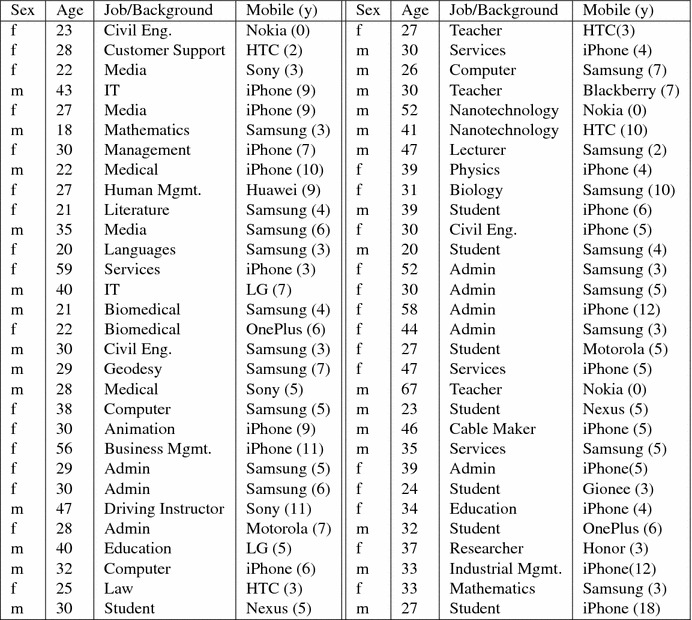

## Appendix 2: Interview script

Hi. Thanks very much for contributing to our study. In this interview, we will ask you to fill in a few questionnaires about mobile sensors such as GPS, camera, light, motion, and orientation. You are encouraged to think out loud as you go through, and please feel free to provide any comments during the interview. There is no right or wrong answer, and our purpose is to evaluate the mobile sensors, not you. Everything about this interview is anonymous. Please provide some information about yourself in Table 8.

### Part one

A list of multiple mobile sensors is presented below. To what extent do you know each sensor on a mobile device? Please rate them in the table (Table 9 was used).

**Table 8:** Demography

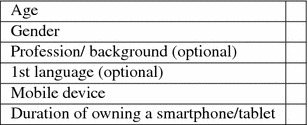

**Table 9:** This form was used for part one

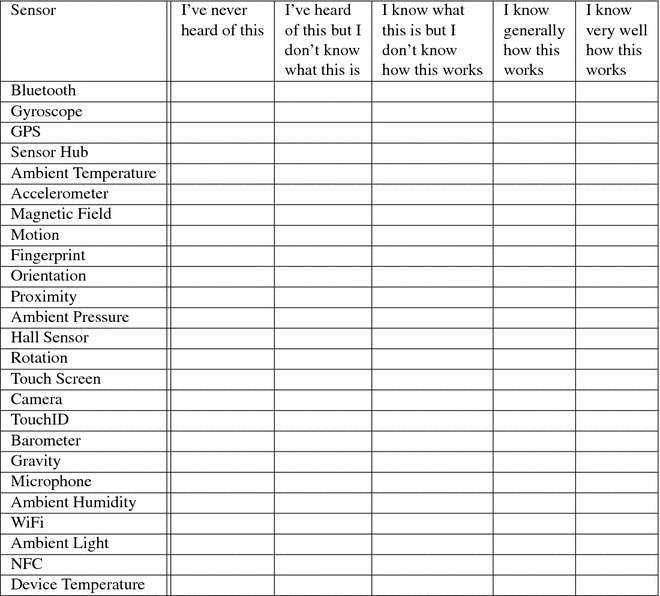

### Part two

Imagine that you own a smartphone which is equipped with all these sensors. Consider this scenario: you have opened a game app which can have access to all mobile sensors. You leave the game app open in the background, and open your banking app which requires you to enter your PIN.

Do you think any of these sensors can help the game app discover your entered PIN? To what extent are you concerned about each sensor’s risk to your PIN? Please rate them in the table (Table 10 was used). In this section, please only rely on the knowledge you already have about the sensors, and if you do not know some of them, describe your feeling of security about them.

**Table 10:** This form was used for parts two and three

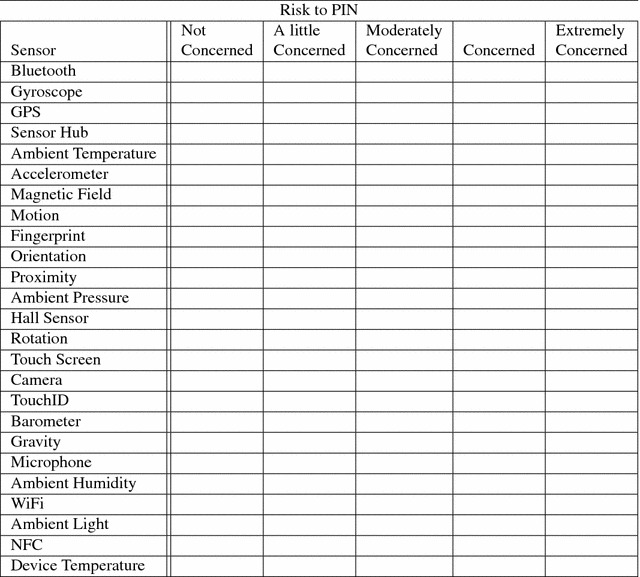

### Part three

Let us explain each sensor here:

- GPS: identifies the real-world geographic location.
- Camera, Microphone: capture pictures/videos and voice, respectively.
- Fingerprint, TouchID: scans the fingerprint.
- Touch Screen: enables the user to interact directly with the display by physically touching it.
- WiFi: is a wireless technology that allows the device to connect to a network.
- Bluetooth: is a wireless technology for exchanging data over short distances.
- Near-Field Communication (NFC): is a wireless technology for exchanging data over shorter distances (less than 10 cm) for purposes such as contactless payment.
- Proximity: measures the distance of objects from the touch screen.
- Ambient Light: measures the light level in the environment of the device.
- Ambient Pressure (Barometer), Ambient Humidity, and Ambient Temperature: measure the air pressure, humidity, and temperature in the environment of the device, respectively.
- Device Temperature: measures the temperature of the device.
- Gravity: measures the force of gravity.
- Magnetic Field: reports the ambient magnetic field intensity around the device.
- Hall sensor: produces voltage based on the magnetic field.
- Accelerometer: measures the acceleration of the device movement or vibration.
- Rotation: reports how much and in what direction the device is rotated.
- Gyroscope: estimates the rotation rate of the device.
- Motion: measures the acceleration and the rotation of the device.
- Orientation: reports the physical angle that the device is held in.
- Sensor Hub: is an activity recognition sensor and its purpose is to monitor the device’s movement.

Please feel free to ask us about any of these sensors for more information.

Now that you have more knowledge about the sensors, let us describe the same scenario here again. Imagine that you own a smartphone which is equipped with all these sensors. You have opened a game app which can have access to all mobile sensors. You leave the game app open in the background, and open your banking app which requires you to enter your PIN.

Do you think any of these sensors can help the game app to discover your entered PIN? To what extent are you concerned about each sensor’s risk to your PIN? Please rate them in the table (Table 10 was used). In this part, please make sure that you know the functionality of all the sensors. If you are unsure, please have another look at the descriptions, or ask us about them.

Thanks very much for taking part in this study. Please leave any extra comment here.

An Amazon voucher and a business card are in this envelope. Please contact us if you have any questions about this interview, or are interested in the results of this study.

## Footnotes

1
http://w3.org/TR/#tr_Javascript_APIs.

2
http://github.com/maryammjd/Reading-sensor-data-for-fifty-4digit-PINs.

3
http://play.google.com/store/apps/details?id=com.solirify.mathgame.

4
http://datagenetics.com/blog/september32012/.

5
http://forums.androidcentral.com/verizon-galaxy-nexus/171482-barometer-accelerometer-how-they-useful.html.

6
http://apple.com/uk/iphone-6/specs/.

7
http://store.google.com/product/nexus_6p.

8
http://w3.org/2009/dap/.

9
http://samsung.com/us/support/answer/ANS00035658/234302/SCH-R950TSAUSC.

10
http://sites.google.com/site/autosenseproject/.

11 Interquartile range.

12
http://w3c.github.io/deviceorientation/spec-source-orientation.html.

13
http://bugs.chromium.org/p/chromium/issues/detail?id=421691.

14
http://mozilla.org/en-US/security/advisories/mfsa2016-43/.

15
http://support.apple.com/en-gb/HT206166.

16
http://bugs.chromium.org/p/chromium/issues/detail?id=523320.

17
http://us.hsbc.com/1/2/home/personal-banking/pib/mobile/touchid.

18
http://hsbc.co.uk/1/2/contact-and-support/banking-made-easy/voice-id.

19
http://brandchannel.com/2015/03/16/alibaba-demos-smile-to-pay-facial-recognition-app/.

20
http://bbc.co.uk/news/technology-32498222.

## References

[1] Google. Location and sensors APIS. http://developer.android.com/guide/topics/sensors/index.html

[2] Jin, X., Hu, X., Ying, K., Du, W., Yin, H., Peri, G.N.: Code injection attacks on HTML5-based mobile apps: characterization, detection and mitigation. In: Proceedings of the 2014 ACM SIGSAC Conference on Computer and Communications Security, CCS ’14, pp. 66–77. ACM, New York (2014)

[3] Kim, H., Lee, S., Kim, J.: Exploring and mitigating privacy threats of HTML5 geolocation API. In: Annual Computer Security Applications Conference (ACSAC). New Orleans (2014)

[4] Mehrnezhad M, Toreini E, Shahandashti SF, Hao F (2016). Touchsignatures: identification of user touch actions and PINs based on mobile sensor data via javascript. J. Inform. Secur. Appl..

[5] Mehrnezhad, M., Toreini, E., Shahandashti, S.F., Hao, F.: Touchsignatures: Identification of user touch actions based on mobile sensors via javascript (extended abstract). In: Proceedings of the 9th ACM Symposium on Information, Computer and Communications Security (ASIACCS 2015). ACM (2015)

[6] W3C Working Draft Document on Device Orientation Event. http://www.w3.org/TR/orientation-event/

[7] Aviv, A.J., Sapp, B., Blaze, M., Smith, J.M.: Practicality of accelerometer side channels on smartphones. In: Proceedings of the 28th Annual Computer Security Applications Conference, pp 41–50. ACM (2012)

[8] Cai, L., Chen, H.: Touchlogger: Inferring keystrokes on touch screen from smartphone motion. In: HotSec (2011)

[9] Cai L, Chen H, Katzenbeisser S, Weippl E, Camp L, Volkamer M, Reiter M, Zhang X (2012). On the practicality of motion based keystroke inference attack. Trust and Trustworthy Computing. Lecture Notes in Computer Science.

[10] Michalevsky, Y., Boneh, D., Nakibly, G.: Gyrophone: recognizing speech from gyroscope signals. In: Proceedings of the 23rd USENIX Security Symposium (2014)

[11] Miluzzo, E., Varshavsky, A., Balakrishnan, S., Choudhury, R.R.: Tapprints: your finger taps have fingerprints. In: Proceedings of the 10th international conference on Mobile systems, applications, and services, pp. 323–336. ACM (2012)

[12] Narain, S., Sanatinia, A., Noubir, G.: Single-stroke language-agnostic keylogging using stereo-microphones and domain specific machine learning. In: Proceedings of the 2014 ACM Conference on Security and Privacy in Wireless and Mobile Networks, WiSec ’14, pp. 201–212. ACM, New York (2014)

[13] Owusu, E., Han, J., Das, S., Perrig, A., Zhang, J.: Accessory: password inference using accelerometers on smartphones. In: Proceedings of the Twelfth Workshop on Mobile Computing Systems and Applications, p. 9. ACM (2012)

[14] Simon, L., Anderson, R.: Pin skimmer: inferring pins through the camera and microphone. In: Proceedings of the Third ACM Workshop on Security and Privacy in Smartphones and Mobile Devices, SPSM ’13, pp. 67–78. ACM, New York (2013)

[15] Spreitzer, R.: Pin skimming: exploiting the ambient-light sensor in mobile devices. In: Proceedings of the 4th ACM Workshop on Security and Privacy in Smartphones and Mobile Devices, SPSM ’14, pp. 51–62. ACM, New York (2014)

[16] Xu, Z., Bai, K., Zhu, S.: Taplogger: inferring user inputs on smartphone touchscreens using on-board motion sensors. In: Proceedings of the fifth ACM conference on Security and Privacy in Wireless and Mobile Networks, pp. 113–124. ACM (2012)

[17] Bichler D, Stromberg G, Huemer M, Löw M, Krumm J, Abowd G, Seneviratne A, Strang T (2007). Key generation based on acceleration data of shaking processes. UbiComp 2007: Ubiquitous Computing. Lecture Notes in Computer Science.

[18] Møller MF (1993). A scaled conjugate gradient algorithm for fast supervised learning. Neural Netw..

[19] Bonneau J, Preibusch S, Anderson R, Keromytis A (2012). A birthday present every eleven wallets? the security of customer-chosen banking pins. Financial Cryptography and Data Security. Lecture Notes in Computer Science.

[20] Al-Haiqi A, Ismail M, Nordin R (2013). On the best sensor for keystrokes inference attack on android. Proced. Technol..

[21] Li, M., Meng, Y., Liu, J., Zhu, H., Liang, X., Liu, Y., Ruan, N.: When CSI meets public WiFi: inferring your mobile phone password via WiFi signals. In: Proceedings of the 2016 ACM SIGSAC Conference on Computer and Communications Security, CCS ’16, pp. 1068–1079. ACM, New York (2016)

[22] Wang, H., Lai, T.T.-T., Roy Choudhury, R.: Mole: motion leaks through smartwatch sensors. In: Proceedings of the 21st Annual International Conference on Mobile Computing and Networking, MobiCom ’15, pp. 155–166. ACM, New York (2015)

[23] W3C Editor’s Draft on Generic Sensor API. http://w3c.github.io/sensors/

[24] Watanabe, T., Akiyama, M., Sakai, T., Mori, T.: Understanding the inconsistencies between text descriptions and the use of privacy-sensitive resources of mobile apps. In: Eleventh Symposium On Usable Privacy and Security (SOUPS 2015), pp. 241–255. USENIX Association, Ottawa (2015)

[25] Kang, R., Dabbish, L., Fruchter, N., Kiesler, S.: “my data just goes everywhere:” user mental models of the internet and implications for privacy and security. In: Eleventh Symposium on Usable Privacy and Security (SOUPS 2015), pp. 39–52. USENIX Association, Ottawa (2015)

[26] Harbach, M., von Zezschwitz, E., Fichtner, A., Luca, A.D., Smith, M.: It’s a hard lock life: a field study of smartphone (un)locking behavior and risk perception. In: Symposium On Usable Privacy and Security (SOUPS 2014), pp. 213–230. USENIX Association, Menlo Park (2014)

[27] De Luca, A., Hang, A., von Zezschwitz, E., Hussmann, H.: I feel like i’m taking selfies all day!: towards understanding biometric authentication on smartphones. In: Proceedings of the 33rd Annual ACM Conference on Human Factors in Computing Systems, CHI ’15, pp. 1411–1414. ACM, New York (2015)

[28] Khan, H., Hengartner, U., Vogel, D.: Usability and security perceptions of implicit authentication: convenient, secure, sometimes annoying. In: Eleventh Symposium on Usable Privacy and Security (SOUPS 2015), pp. 225–239. USENIX Association, Ottawa (2015)

[29] Balebako, R., Jung, J., Lu, W., Cranor, L.F., Nguyen, C.: “Little brothers watching you:” raising awareness of data leaks on smartphones. In: Symposium on Usable Privacy and Security. ACM: Association for Computing Machinery (2013)

[30] Raij, A., Ghosh, A., Kumar, S., Srivastava,M.: Privacy risks emerging from the adoption of innocuous wearable sensors in the mobile environment. In: Proceedings of the SIGCHI Conference on Human Factors in Computing Systems, CHI ’11, pp. 11–20. ACM, New York (2011)

[31] Bojinov, H., Michalevsky, Y., Nakibly, G., Boneh, D.: Mobile device identification via sensor fingerprinting. *CoRR*, abs/1408.1416 (2014)

[32] Das, A., Borisov, N., Caesar, M.: Exploring ways to mitigate sensor-based smartphone fingerprinting. *CoRR*, abs/1503.01874 (2015)

[33] Bravo-Lillo, C., Komanduri, S., Cranor, L.F., Reeder, R.W., Sleeper, M., Downs, J., Schechter, S.: Your attention please: Designing security-decision UIs to make genuine risks harder to ignore. In: Proceedings of the Ninth Symposium on Usable Privacy and Security, SOUPS ’13, pp. 6:1–6:12. ACM, New York (2013)

[34] Charland, A., Leroux, B.: Mobile application development: Web vs. native. Commun. ACM **54**(5), 49–53 (2011)

[35] Enck, W., Gilbert, P., Han, S., Tendulkar, V., Chun, B.-G., Cox, L.P., Jung, J., McDaniel, P., Sheth, A.N.: Taintdroid: an information-flow tracking system for realtime privacy monitoring on smartphones. In: Transactions on Computer Systems (2014)

